# An Unusual Case of Unilateral Epistaxis: The Hidden Bloodsucker

**DOI:** 10.7759/cureus.34417

**Published:** 2023-01-30

**Authors:** Nadhirah Mohd Shakri, Mimi Ezreena Esa

**Affiliations:** 1 Otolaryngology - Head and Neck Surgery, Hospital Tengku Ampuan Rahimah, Klang, MYS

**Keywords:** leech infestation, nasal bleed, hemorrhage, nasal foreign body, primary care, endoscopic, leeches, recurrence, epistaxis

## Abstract

Nasal leech infestation is a rare etiology of epistaxis. Due to its insidious presentation and inconspicuous site of infestation, it is possible for the primary care setting to miss the diagnosis. We report a case of nasal leech infestation in an eight-year-old male child who was repeatedly treated for upper respiratory infection before finally being referred to the otorhinolaryngology clinic. We emphasize the importance of having a high index of suspicion and thorough history taking, especially of jungle trekking and hill water exposure in unexplained recurrent epistaxis.

## Introduction

Nasal foreign bodies have been widely reported in the otorhinolaryngology (ORL) literature with unilateral nasal discharge in children aged 1-5 years being the most common presentation [1]. However, leech infestation in the nose causing unilateral epistaxis is rare and scarcely reported. Leeches have also been found in the pharynx, larynx, and trachea leading to serious complications such as upper airway obstruction and severe anemia, requiring blood transfusion [2,3]. A thorough history taking, especially of jungle trekking and hill water or river exposure, plays a vital role in alerting the physicians about the diagnosis and preventing further morbidities [4].

## Case presentation

**Figure 1:**
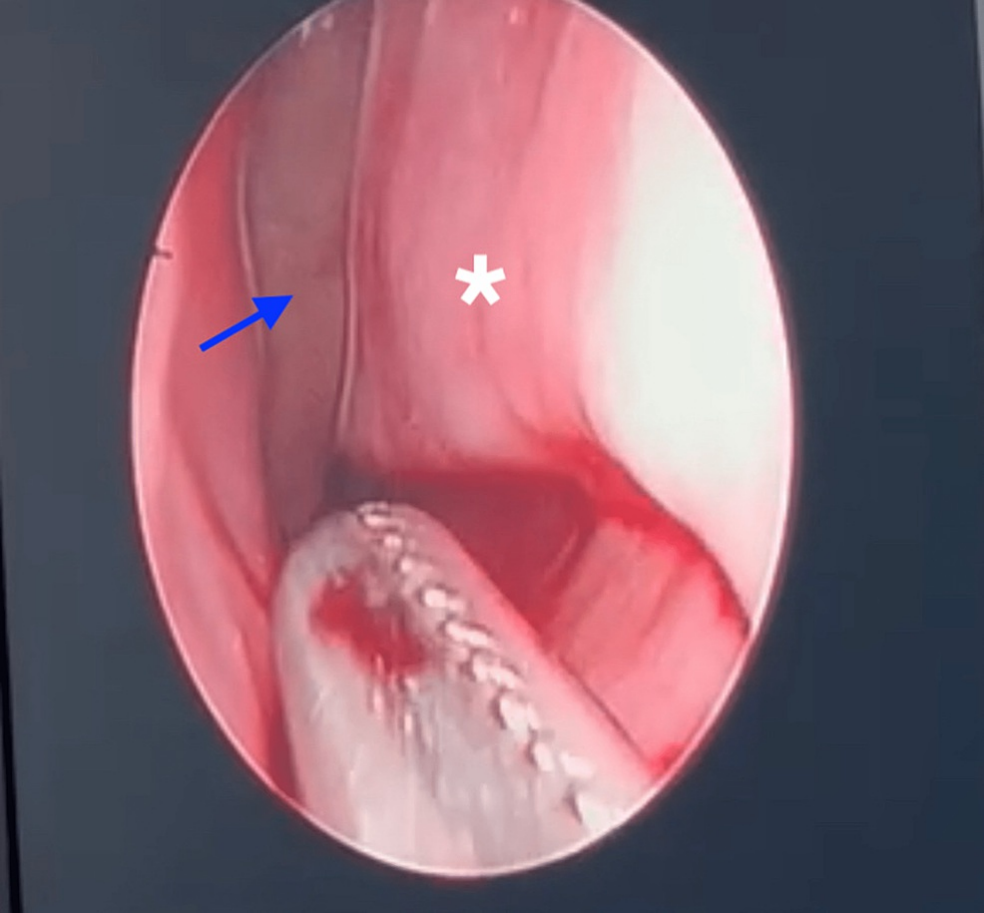
Nasal endoscopic view of left nasal cavity Nasal endoscopic examination revealed a leech (blue arrow) located at the posterior end of left inferior turbinate (asterisk). The leech almost migrated posteriorly to the nasopharynx during the procedure; however, it was grasped and removed using Tilley’s forceps.

**Figure 2:**
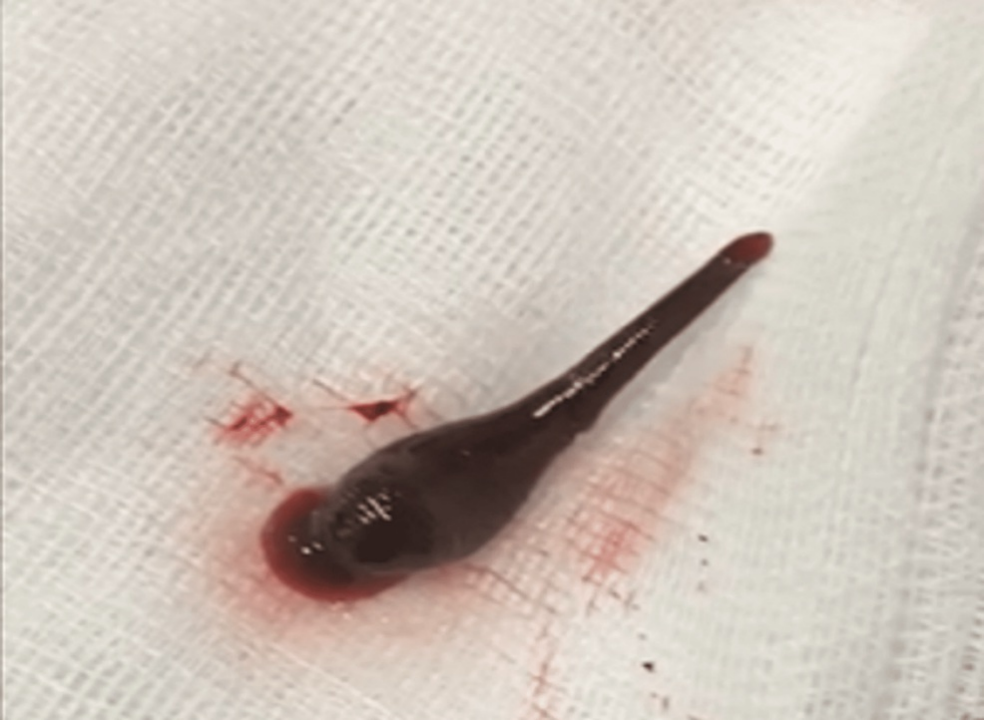
The leech after removal The leech was still alive after removal, measuring 4 centimeters in length.

An eight-year-old boy suffered from recurrent unilateral epistaxis for three weeks, which soaked the handkerchief in small drops. He denied having other nasal symptoms such as nasal blockage, itchiness, or frequent sneezing. He did not complain of fever or foul-smelling nasal discharge that may suggest infection. He sought treatment at three different clinics where he was treated with antibiotics and anti-histamine before finally being referred to our ORL clinic for further assessment due to the persistence of symptoms. A thorough history revealed that he had a history of swimming in a river in a rural area, which was his mother's hometown. Nasoendoscopic examination after topical anesthesia (co-phenylcaine nasal spray) revealed a living leech at the posterior end of the left inferior turbinate (Figure 1). After a few attempts, the leech was successfully removed (Figure 2) using Tilley’s forceps. Repeated nasoendoscopy was performed to look for any active bleeding, residual leech, and other causes of epistaxis. No bleeding from nasal mucosa was observed after the procedure. 

## Discussion

Leeches are annelids that may infest the human body via various orifices during exposure to hill water or river, commonly encountered in rural areas [4]. Once attached to the host’s mucous membrane, the leech will release anticoagulant hirudin and histamine-like vasodilator to assist in feeding and interfere with the coagulation process, leading to hemorrhage [5]. Its saliva contains enzymes that will anesthetize the attachment site leading to an absence of pain, and ensuring the host's unawareness of its presence [5]. As a result, delay of treatment is not uncommonly attributable to the unostentatious presentation.

Presentation varies depending on the duration and site of infestation. The most common presentation for nasal leech infestation is recurrent painless unilateral epistaxis [5]. Leeches have also been found in the pharynx, larynx, and trachea presenting with hemoptysis and blood-stained saliva [2,3,6]. Although rare, complications such as upper airway obstruction and severe anemia requiring blood transfusion have been reported in the literature [2,3,7]. Tilahun reported an unusual case of vaginal leech infestation complicated with hypovolemic shock in a postmenopausal woman [7].

Patients commonly seek treatment at primary care centers where facilities are limited. Due to the inconspicuous site of infestation, leeches are not usually visualized by bedside physical examinations such as anterior rhinoscopy. Furthermore, active hemorrhage may further obscure the view. Thus, it is paramount to have a thorough history taking and a high index of suspicion before an immediate referral to ORL is made. Urgent removal of leeches may prevent complications and further morbidities. 

The removal of a nasal leech is mostly straightforward using forceps after administering local anesthesia (lidocaine) to paralyze the leech. In a cooperative patient, it is often performed endoscopically in an outpatient setting without significant post-removal hemorrhage. However, in certain cases when the leeches are firmly attached to the host, extra maneuvers may be necessary. Chen et al. reported giving electric shock using bipolar diathermy before pulling out a leech with forceps [8]. Other methods include saline irrigation and disintegrating the leeches [4,8]. Leeches may also migrate during the procedure, requiring removal via the oral cavity [3]. Airway leech infestation in the larynx and trachea often requires direct laryngoscopy and removal under general anesthesia [2,3,6]. Repeated endoscopic examination and meticulous inspection post removal are mandatory to assess for hemorrhage and to look for other pathology such as tumors and the possibility of multiple leeches [8]. 

## Conclusions

Unexplained recurrent epistaxis should raise a suspicion of nasal leech infestation despite the patient living in the city area. Thorough history taking, especially that of traveling to rural areas, jungle trekking, and hill water exposure is paramount to get to the diagnosis. Primary care plays a significant role in referring patients to ORL clinics where further assessment and treatment can be given urgently. Removal of leeches is often done in outpatient settings; however, in certain cases, with airway leech infestation or patients who are not cooperative, may require assessment and removal under general anesthesia. Early referral and intervention may prevent further complications.
